# Endoscopic Removal of an Intrabronchial Foreign Body Using Single-Use Flexible Bronchoscopy in an Older Adult: A Case Report and Review of the Literature

**DOI:** 10.7759/cureus.115850

**Published:** 2026-09-06

**Authors:** Amina Betkaoui, Sylvain Marchand-Adam, Belkacem Mansour, Anna Bourreau, Laurent Vaz, Antoine Levy

**Affiliations:** 1 Pulmonology Department, Centre Hospitalier Jacques Coeur, Bourges, FRA; 2 Pulmonology Department, Centre Hospitalier Régional Universitaire, Tours, FRA; 3 Hematology-Oncology Department, Centre Hospitalier Jacques Coeur, Bourges, FRA; 4 Clinical Research and Intensive Care Department, Centre Hospitalier Jacques Coeur, Bourges, FRA; 5 Intensive Care Department, Centre Hospitalier Jacques Coeur, Bourges, FRA

**Keywords:** acute hypoxic respiratory distress, emergency bronchoscopy, flexible bronchoscopy, foreign body aspiration, older adult

## Abstract

Foreign body aspiration (FBA) is less common in adults than in children and occurs predominantly in older individuals with predisposing risk factors. Depending on the anatomical location of the foreign body within the tracheobronchial tree, it may cause life-threatening airway obstruction or lead to a wide range of clinical manifestations and complications, the occurrence of which largely depends on the duration of impaction.

We report the case of a 74-year-old woman who was admitted with acute respiratory distress following the accidental aspiration of a solid food bolus. The initial choking episode resolved spontaneously but subsequently progressed to complete obstruction of the left main bronchus. The foreign body was successfully removed using a single-use flexible bronchoscope (Ambu® aScope 4 Broncho; Ambu A/S, Denmark) approximately one hour after emergency department admission and six hours after the patient's last meal.

This case highlights the safety and effectiveness of flexible bronchoscopy for the emergency removal of tracheobronchial foreign bodies. It also underscores the clinical value of single-use flexible bronchoscopy when rigid bronchoscopy is unavailable, allowing prompt management while avoiding the need for interhospital transfer to a tertiary care center.

## Introduction

Foreign body aspiration (FBA) into the tracheobronchial tree is a rare event in adults, accounting for approximately 0.16%-0.33% of respiratory emergencies [1]. In older adults, the risk of FBA is increased by several predisposing factors, including dysphagia, poor dentition, neurological or psychiatric disorders, impaired protective airway reflexes, and the use of sedative medications [2,3]. Depending on the location and degree of airway obstruction, FBA may present as a life-threatening emergency requiring prompt diagnosis and intervention. 

Bronchoscopy remains the cornerstone of both the diagnosis and management of tracheobronchial foreign bodies. Although rigid bronchoscopy has traditionally been considered the standard therapeutic approach, flexible bronchoscopy has emerged as a safe and effective alternative in selected adult patients, particularly when performed by experienced operators [4].

We report the case of an elderly woman presenting with complete obstruction of the left main bronchus caused by the aspiration of a solid food fragment. The foreign body was successfully removed in an emergency setting using a single-use flexible bronchoscope (Ambu® aScope 4 Broncho; Ambu A/S, Denmark). 

We discuss the diagnostic and therapeutic management of this case in light of the current literature, with particular emphasis on the potential role of single-use flexible bronchoscopy when rigid bronchoscopy is not readily available.

## Case presentation

A 74-year-old woman residing in a long-term care facility was transported to the emergency department by the mobile intensive care unit (MICU) because of acute respiratory distress. Her medical history was significant for essential hypertension and depressive disorder.

The patient reported an initial choking episode during her last meal after accidentally aspirating a piece of meat. Although the choking episode resolved spontaneously, it was followed by the abrupt onset of severe dyspnea (New York Heart Association (NYHA) functional class IV) associated with an ineffective cough [1]. On arrival, she was conscious, afebrile, and hemodynamically stable but exhibited marked tachypnea and severe hypoxemia, with an oxygen saturation of 60% on room air.

Oxygen therapy was initiated using a non-rebreather mask at 15 L/min and was rapidly escalated to high-flow nasal oxygen (HFNO), resulting in respiratory stabilization. Physical examination revealed markedly decreased breath sounds and wheezing over the left hemithorax, whereas breath sounds were preserved over the right lung field. 

A frontal chest radiograph (Figure 1) demonstrated complete left lung atelectasis associated with ipsilateral mediastinal shift and compensatory hyperinflation of the right lung.

**Figure 1:**
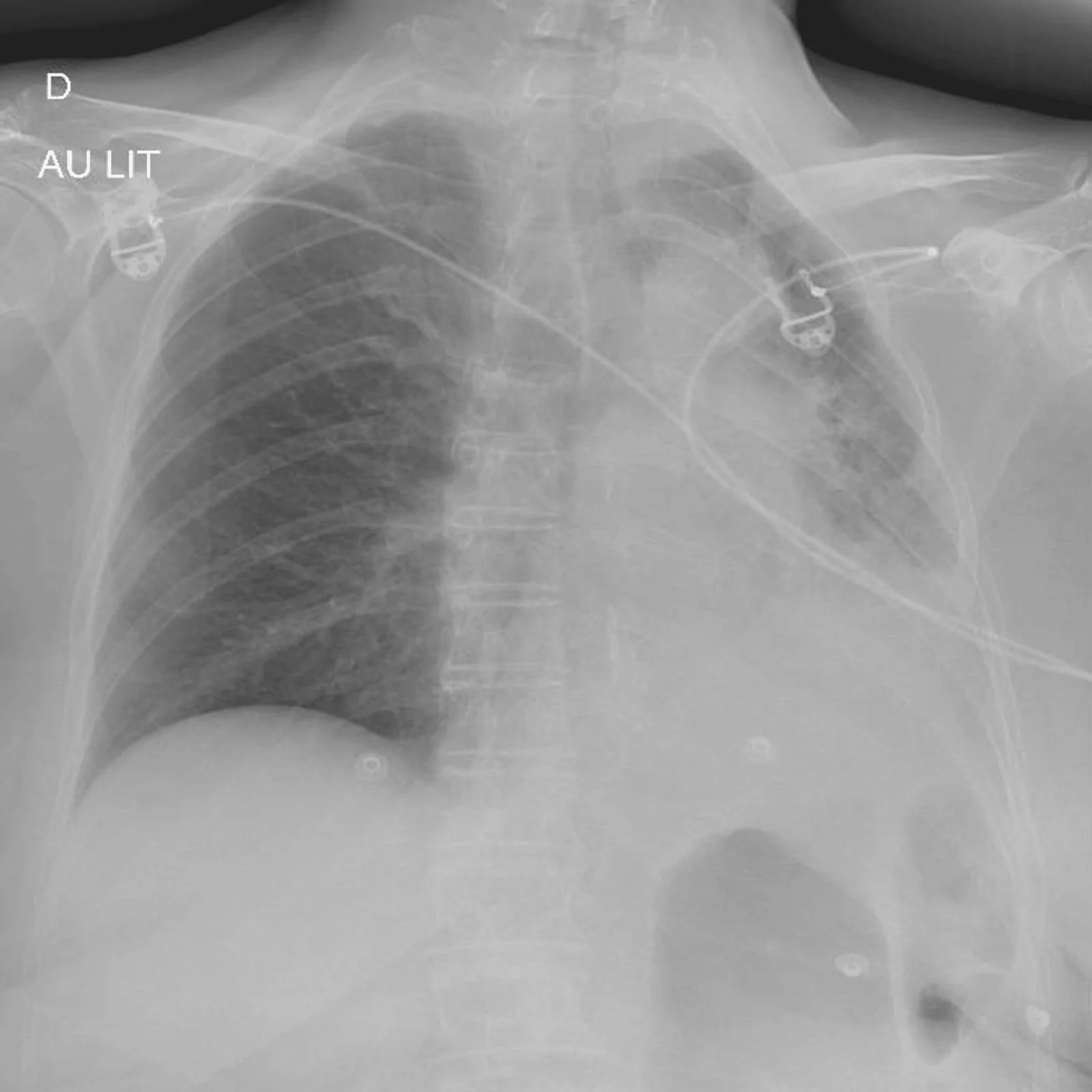
Frontal chest radiograph demonstrating complete left lung atelectasis associated with ipsilateral mediastinal shift.

Given the patient's hemodynamic and respiratory stability under HFNO and the absence of rigid bronchoscopy facilities at our institution, urgent transfer to the nearest tertiary referral center, located 172 km away (approximately a two-hour journey), would have resulted in a clinically significant delay. Considering the need for prompt intervention, a single-use flexible bronchoscopy was performed 45 minutes after admission to the emergency department and approximately six hours after the patient's last meal.

Bronchoscopic examination revealed a solid food fragment firmly impacted within the left main bronchus, resulting in complete airway obstruction. The surrounding bronchial mucosa appeared inflamed, with abundant whitish secretions. Examination of the remaining tracheobronchial tree revealed no additional abnormalities (Figure 2).

**Figure 2:**
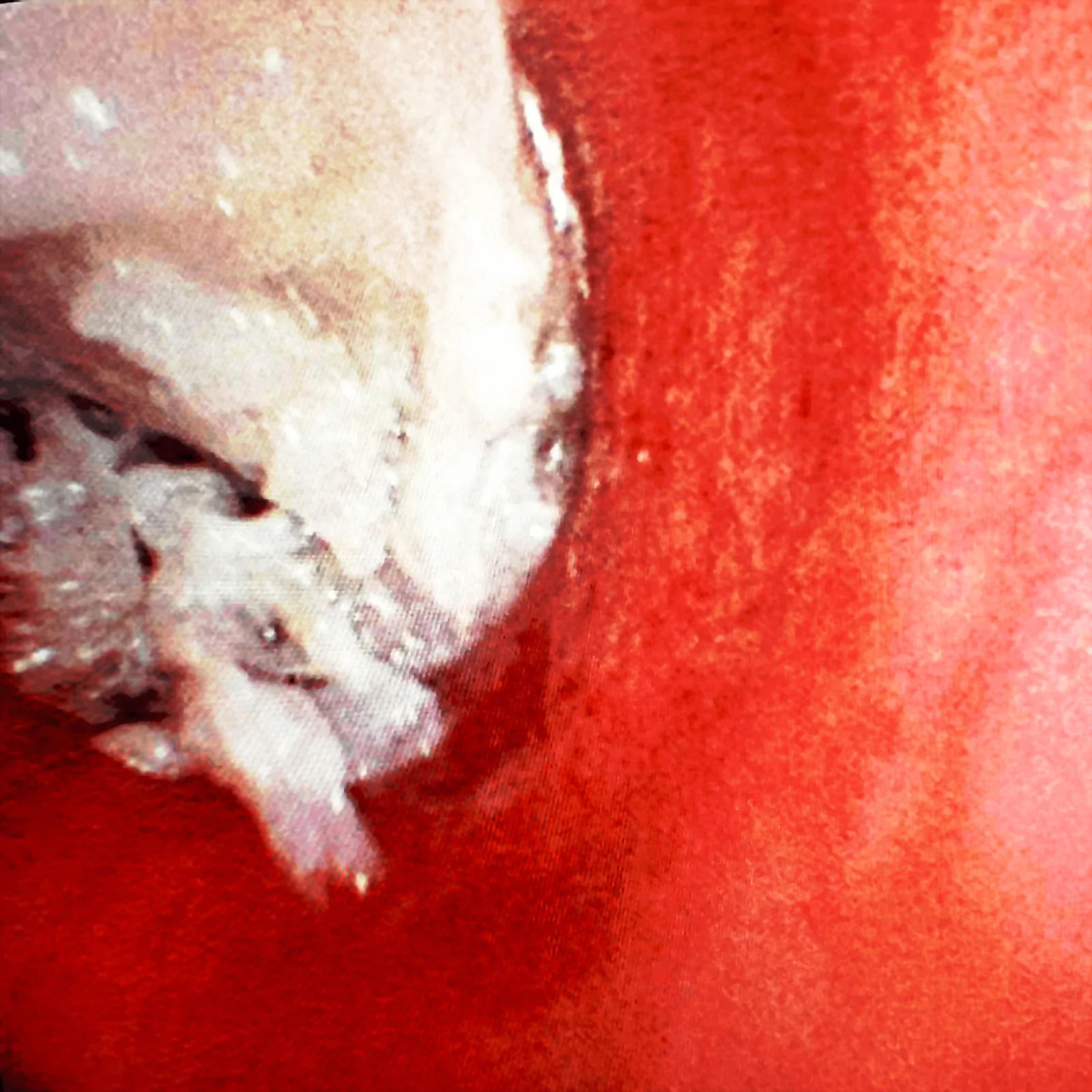
Bronchoscopy showing a foreign body causing complete obstruction of the left main bronchus.

The foreign body was removed using crocodile forceps (Olympus EndoJaw 2.0 mm; Olympus Medical Systems). Several attempts were required to dislodge the impacted fragment from the left main bronchus. Once mobilized, continuous suction maintained the foreign body at the tip of the bronchoscope, allowing successful en bloc extraction. The retrieved foreign body consisted of a whitish, soft food fragment measuring approximately 1.0 × 1.5 cm.

Follow-up bronchoscopy confirmed complete restoration of left main bronchial patency, with residual inflammatory changes of the bronchial mucosa (Figure 3). 

**Figure 3:**
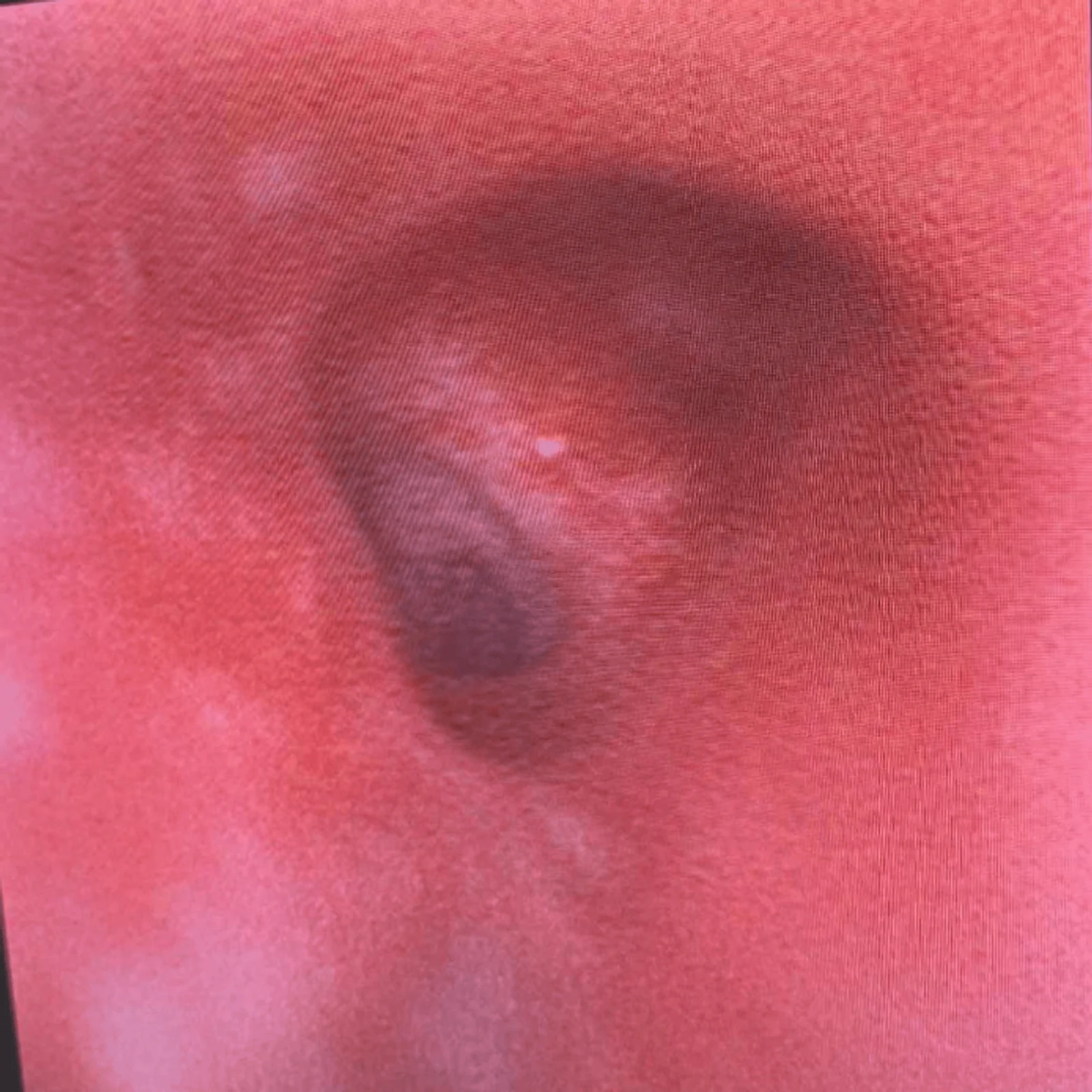
Post-extraction findings. The bronchus was patent, with mildly inflamed mucosa.

The patient's clinical course was immediately favorable. Oxygen requirements rapidly decreased, dyspnea resolved completely, and follow-up chest radiography (Figure 4) demonstrated full re-expansion of the left lung. She was discharged on hospital day 7 without procedure-related or respiratory complications.

**Figure 4:**
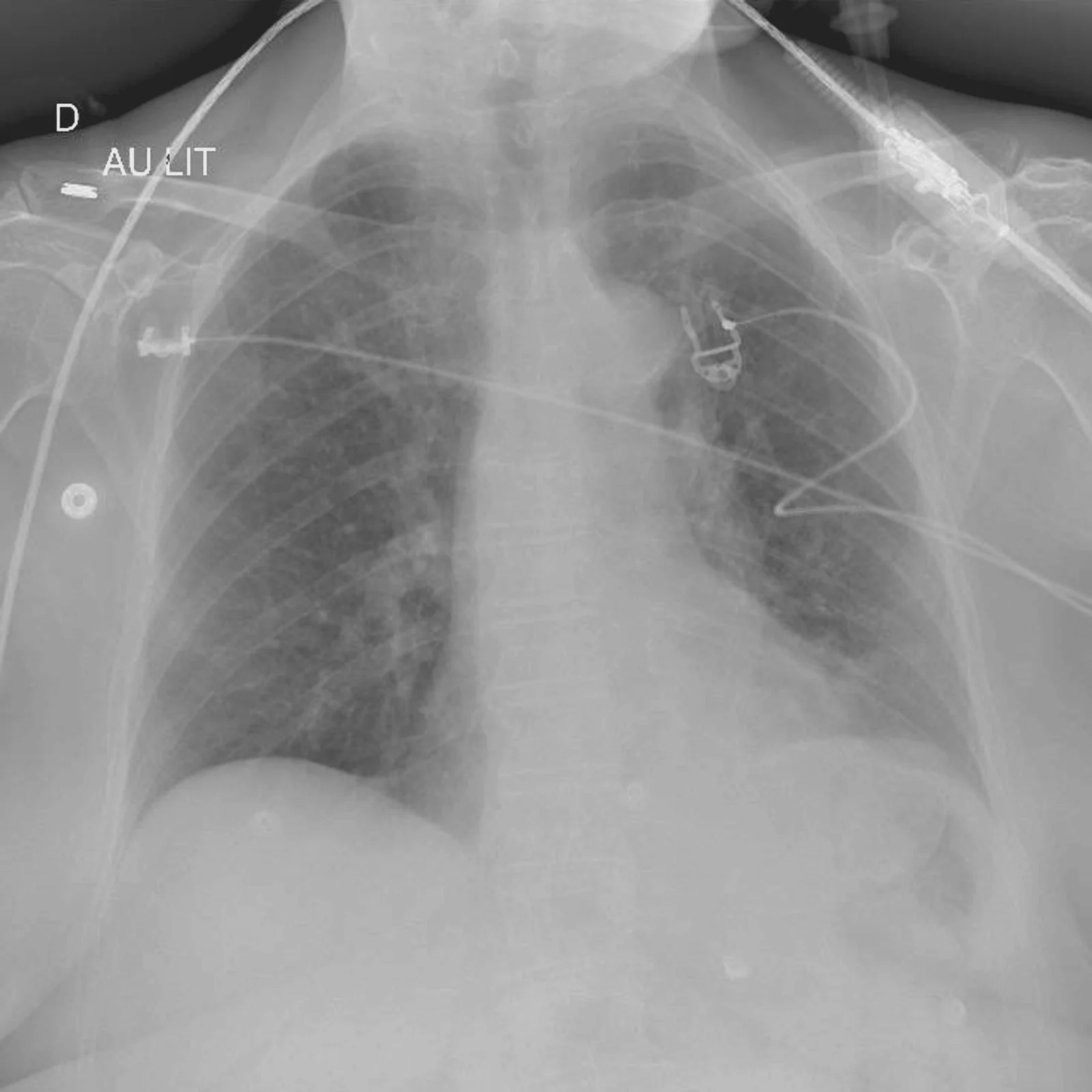
Post-extraction posteroanterior chest radiograph showing removal of the foreign body.

## Discussion

In older adults, FBA is facilitated by several well-established risk factors, including swallowing disorders [1,2], which are frequently underdiagnosed, cognitive or psychiatric disorders [3], age-related attenuation of the cough reflex, the use of sedative or psychotropic medications, and poor dentition. These factors contribute to the occurrence of aspiration events, which may be severe and present with acute respiratory distress, as observed in the present case.

Comparison of our case with the findings reported in the literature (Table 1) highlights several points of interest. First, the age of our patient (74 years) is consistent with the geriatric population commonly affected by this type of event, similar to the cases reported in the literature [4]. The type of foreign body identified in our patient, namely a food fragment, is also consistent with previously reported findings in older adults, in whom food particles, dentures, and bone fragments are predominant, in contrast to metallic objects, which are more frequently encountered in younger adults [5,6].

**Table 1:** Comparison of the present case with previously reported cases.

Study	Study population/sample size	Foreign body characteristics	Bronchoscopic approach	Main findings/success rate	Complications/outcome
Limper and Prakash (1990) [1]	Adults with tracheobronchial foreign bodies	Various organic and inorganic foreign bodies	Bronchoscopy	Bronchoscopic removal was successful in the majority of cases	Overall favorable outcomes; complications were uncommon
Lan (1994) [2]	47 adults	Bone was the most common foreign body in chronic and uncertain cases	Bronchoscopy	Clinical presentation and management varied according to the duration of foreign body retention	Delayed diagnosis was associated with chronic respiratory manifestations
Chen et al. (1997) [3]	43 adults	Various aspirated foreign bodies; organic material was common	Bronchoscopy	Foreign body aspiration was frequently associated with nonspecific respiratory symptoms	Choking was reported in only a minority of patients; delayed diagnosis was common
Debeljak et al. (1999) [4]	62 adults	Various tracheobronchial foreign bodies	Bronchoscopy	Bronchoscopic extraction was effective in most patients	Favorable clinical outcomes with a low rate of complications
Mise et al. (2009) [7]	Adults with tracheobronchial foreign bodies (1995–2006)	Various foreign bodies	Flexible bronchoscopy	Flexible bronchoscopy was effective for foreign body extraction in adults	Most foreign bodies were successfully removed without major complications
Ramos et al. (2009) [8]	Adults with airway foreign bodies (1987–2008)	Predominantly organic foreign bodies	Flexible and/or rigid bronchoscopy	Bronchoscopic extraction was successful in the majority of cases	Surgical intervention was reserved for unsuccessful bronchoscopic extraction or irreversible complications
Sehgal et al. (2015) [9]	65 patients; systematic review of 1,554 adults	Metallic (41%) and organic (25.6%) foreign bodies in their institutional series	Flexible bronchoscopy	Foreign bodies were successfully removed in 45/49 cases (91.8%); pooled success rate of flexible bronchoscopy was 89.6%	No major complications in the institutional series
Fang et al. (2015) [10]	94 adults	Teeth and bone were the most common foreign bodies	Flexible bronchoscopy with multiple extraction modalities	Flexible bronchoscopy successfully removed foreign bodies in 90% (85/94) of patients	Nine procedures failed; eight patients subsequently underwent rigid bronchoscopy and one underwent lobectomy for lung abscess
Ramos et al. (2016) [5]	Literature review	Organic foreign bodies, particularly bone fragments and seeds, were most common	Flexible and rigid bronchoscopy	Flexible bronchoscopy can safely remove most airway foreign bodies in adults	Surgery should generally be reserved for failed bronchoscopic extraction or irreversible bronchial/lung complications
Sancho-Chust et al. (2020) [6]	32 adults with airway foreign bodies among 12,588 bronchoscopies	85% organic; animal material 57% and vegetable material 28%	Flexible bronchoscopy	Successful removal in 94% of cases; rigid bronchoscopy was required in 6%	No foreign-body-related complications; right bronchial tree was the most common location (69%)
Present case (2025)	74-year-old woman	Meat fragment	Single-use flexible bronchoscopy	Successful emergency extraction with complete re-expansion of the left lung	No procedure-related complications; discharged on day 7

Although the right bronchial tree is classically the most common site of FBA because of the more vertical orientation of the right main bronchus, left-sided bronchial involvement has also been described [7], particularly when the foreign body is large or has been aspirated deeply [8]. However, this localization remains uncommon [9], reported in approximately 5.7% of cases, as observed in our patient [10].

Regarding management, the timing of bronchoscopy should be determined according to the patient's clinical status and respiratory tolerance. In patients presenting with signs of respiratory distress, bronchoscopy should be performed urgently. In this context, training pulmonologists, emergency physicians, intensivists, and anesthesiologists in the use of single-use flexible bronchoscopes may be particularly valuable when rigid bronchoscopy is not immediately available. In our case, endoscopic removal of the foreign body resulted in complete pulmonary re-expansion and rapid clinical recovery without complications, thereby avoiding the need for medical evacuation. These findings are consistent with the outcomes reported in the literature [5,6].

Our case highlights the importance of rapid diagnosis and timely, appropriate endoscopic management, which can lead to a favorable outcome even in patients presenting with severe initial respiratory compromise.

## Conclusions

Single-use flexible bronchoscopy appears to be a safe and well-tolerated technique for the removal of non-sharp bronchial foreign bodies in older adults. It may represent an effective alternative when rigid bronchoscopy is not immediately available. Therefore, training pulmonologists, intensivists, emergency physicians, and anesthesiologists in the use of single-use flexible bronchoscopes should be encouraged, particularly in settings where access to rigid bronchoscopy is limited or delayed.
